# The Significance of Interferon-γ in HIV-1 Pathogenesis, Therapy, and Prophylaxis

**DOI:** 10.3389/fimmu.2013.00498

**Published:** 2014-01-13

**Authors:** Shannon R. Roff, Ezra N. Noon-Song, Janet K. Yamamoto

**Affiliations:** ^1^Department of Infectious Diseases and Pathology, College of Veterinary Medicine, University of Florida, Gainesville, FL, USA

**Keywords:** interferon-γ, HIV-1 pathogenesis, IFNγ therapy, anti-retroviral therapy, HIV-1 vaccine

## Abstract

Interferon-γ (IFNγ) plays various roles in the pathogenesis of HIV/AIDS. In an HIV-1 infected individual, the production of IFNγ is detected as early as the acute phase and continually detected throughout the course of infection. Initially produced to clear the primary infection, IFNγ together with other inflammatory cytokines are involved in establishing a chronic immune activation that exacerbates clinical diseases associated with AIDS. Unlike Type 1 IFNs, IFNγ has no direct antiviral activity against HIV-1 in primary cultures, as supported by the *in vivo* findings of IFNγ therapy in infected subjects. Results from both *in vitro* and *ex vivo* studies show that IFNγ can instead enhance HIV-1 replication and its associated diseases, and therapies aimed at decreasing its production are under consideration. On the other hand, IFNγ has been shown to enhance cytotoxic T lymphocytes and NK cell activities against HIV-1 infected cells. These activities are important in controlling HIV-1 replication in an individual and will most likely play a role in the prophylaxis of an effective vaccine against HIV-1. Additionally, IFNγ has been used in combination with HIV-1 vaccine to augment antiviral immunity. Technological advancements have focused on using IFNγ as a biological marker to analyze the type(s) of immunity generated by candidate HIV vaccines and the levels of immunity restored by anti-retroviral drug therapies or novel immunotherapies. Hence, in addition to its valuable ancillary role as a biological marker for the development of effective HIV-1 prophylactic and therapeutic strategies, IFNγ has a vital role in promoting the pathogenesis of HIV.

## Interferon-γ in the Pathogenesis of HIV-1

Interferon-γ (IFNγ) is a Type II interferon that is pivotal in the regulation of the host immune response against viral and intracellular bacterial pathogens. The effects of IFNγ are broad and far-reaching, exhibiting polyfunctional effects on immune activation, proinflammatory responses, and immune modulation. High levels of IFNγ are secreted by Type 1 T helper cells (Th1 cells), CD8^+^ cytotoxic T lymphocytes (CD8^+^ CTLs), and NK cells during active infection (1). IFNγ has a major effect on the regulation of antigen presentation by macrophages and dendritic cells, and in induction of class switching of B cells (2, 3). As a proinflammatory cytokine IFNγ directly activates phagocytic cells and stimulates oxidative burst and the release of degradative enzymes, thereby supporting the host defense responses against intracellular pathogens (4). IFNγ also induces the production of proinflammatory cytokines and chemokines on endothelial cells, epithelial cells, and fibroblasts. Focal release of IFNγ results in vasodilation and upregulation of adhesion molecules, promoting diapedesis of neutrophils, and macrophages to the site of inflammation. In addition to upregulation of innate defense mechanisms, IFNγ is also pivotal in immune modulation. Moreover, IFNγ upregulates the expression of MHC-I and -II molecules, activates antigen presenting cells and induces macrophage maturation toward a proinflammatory phenotype (4, 5). IFNγ also works synergistically with other cytokines such as IL-2 and -4 to balance the T helper subsets Th1/Th2, regulating the cytotoxic versus humoral T cell immune response (6). It is this integral part in the immune regulation and proinflammatory antiviral response that has made IFNγ an attractive biomarker to evaluate the immune competence and antiviral response in HIV-1 patients.

### IFNγ expression in seronegative infants breastfed by HIV-1 positive mothers

It is estimated that 20–45% of infants born to HIV positive mothers become HIV positive in the perinatal period (7, 8). Of these infants, 25–35% infected within the first year of life acquire the disease through breast feeding (7). Although this patient subset represents individuals with naïve and underdeveloped immune systems, multiple studies have demonstrated competent HIV-1 specific IFNγ responses in infants <1 year of age (7–11). Legrand et al. detected HIV-1 specific IFNγ responses generated specifically from CD8^+^ T cell subsets, demonstrating the ability of this naïve immune system to develop an antigen-specific T cell response (11). In a Nairobi trial of more than 200 breast fed infants born to HIV-1 positive (HIV^+^) mothers, more than half of exposed infants remained seronegative for the first year of life (8). The IFNγ response in these exposed but uninfected infants was significantly increased compared with infected cohorts, revealing a positive correlation of increased IFNγ response with those infants that remained HIV-1 seronegative up to 1 year of age (Table 1) (8). Although there was a significant increase in IFNγ expression in the exposed/uninfected infants, IFNγ levels were moderate and often cyclical to transient with only 12–22% of them having detectable IFNγ at any given time point (8). This suggests that prolonged, repeated exposure to HIV-1 through breast feeding was a significant factor in inducing and maintaining an IFNγ response (8, 9). Highly exposed, persistently seronegative sex workers demonstrate high levels of HIV-1 specific IFNγ responses in CD8^+^ CTLs (Table 1) (12). Individuals within this cohort who had a 2-month or greater break in sex work were 6.5 times more likely to seroconvert, suggesting a significant decrease in HIV-1 antigen-specific response resulting from a gap in prolonged and repeated antigen exposure (12).

**Table 1:** **Predictive value of IFNγ expression**.

Disease status	Age	HIV-specific IFNγ expression	Predictive value of IFNγ expression	Reference
Highly exposed, seronegative	Breastfed infant	Elevated, transient	Positive correlation with seronegative status	(8)
Highly exposed, seronegative	Adult	Highly elevated	No correlation to rate of seroconversion	(12)
Infected	Infant	Elevated, attenuated compared to adults	No correlation with HIV load set points or mortality	(7, 8)
Acute	Adult	Elevated	No correlation with HIV load set point or disease progression	(13)
Chronic, non-progressive	Adult	Persistently elevated	No correlation with stage or chronicity of clinical disease	(14–16)
Chronic, progressive	Adult	Persistently elevated	No correlation with stage or chronicity of clinical disease	(14–16)

### IFNγ expression in HIV-1 infected neonates and infants

Although the naïve state of the neonatal and infant immune system is a concern in HIV-1 exposure, HIV-1 specific CTL responses have been reported in both exposed and infected infants (7, 8, 10, 11). HIV-1 infected infants <1 year of age with a detectable circulating viral load mount a substantial and sustained antigen-specific immune response. However, the magnitude of their response is attenuated as compared with adults (8). It is theorized that this attenuated response in infected infants may be attributed to an overall decrease in IFNγ producing cells, a suppression of the Th1 response, underdevelopment of the CD4^+^ T cell repertoire, or immaturity of antigen processing related to age (10). Although a reduction of the number of IFNγ producing cells correlates with a decrease in the overall CD4^+^ counts in infected infants a twofold increase in HIV-1 specific IFNγ response was detected in infected infants in the first year of life (10). This increase not only demonstrates a continued expression of IFNγ in infected infants, but also showed a significant trend toward an increased immunologic response when limited to those breastfed infants surviving 1 year or more (10). Even though an increased IFNγ response and HIV-1 specific CTL can be detected in HIV-1 infected infants, there is no correlation between the presence of antigen-specific CTL or IFNγ response to reduction in peak viral load, viral steady state, or incidence of mortality in infected infants (Table 1) (10). Therefore, this positive trend likely reflects the immunologic stimulation attributed to continued and prolonged exposure to HIV-1 rather than a sustained response to initial infection.

### IFNγ expression in acute and chronic HIV-1 infection

Throughout the acute stage of HIV-1 infection, IFNγ levels in infected adults steadily increase, with a peak approximately 20–24 days post-infection (Table 1) (13). In chronic, stable disease, IFNγ levels decline to a steady state that is often equivalent to healthy controls (17). Although there is a predictable elevated trend in overall IFNγ expression during HIV-1 clinical disease, no significant difference has been reported in HIV-1 specific IFNγ response of the CTLs in both progressor and long-term non-progressor patients with chronic disease (17). Although overall expression of IFNγ by CD8^+^ CTLs does not correlate to stage or chronicity of clinical disease, significantly larger numbers of HIV-1 specific CTLs are maintained in long-term non-progressors (18, 19). There is a significant trend of steady increase of IFNγ levels in chronic, progressive disease however, there is marked patient to patient variability in overall expression of IFNγ with no demonstrable correlation between IFNγ expression and viral load, viral set point, viral clearance, or chronicity (14–16) (Table 1). In a cross-sectional study performed by Wantanabe et al. (20), proinflammatory cytokines TNF-alpha, IL-6, IL-10, IL-18, and IL-7 levels had a significant correlation with CD4 count in HIV^+^ patients, but IFNγ levels were often continuously elevated and variable between patients with no significant correlation to progressors and long-term non-progressors in chronically infected patients (20). Several theories exist as to why IFNγ response does not correlate with disease progression and largely center on the polyfunctional and proinflammatory effects of IFNγ (16). It has been suggested that cytokine expression and immunologic profiles in HIV^+^ patients are more proinflammatory than immunoregulatory when compared to uninfected but exposed controls (21). It is also likely that HIV-1 infection results in modification of antigen presentation in macrophages and dendritic cell lines, resulting in anergy of HIV-1 specific CD4^+^ and CD8^+^ T cells (22). Another theory suggests diminished response to IFNγ in target populations may alter immunomodulation of Th1/Th2 response through the production of synergistic (IL-2, TNFα) or inhibitory (TGFβ, IL-10, IL-13) cytokines (16). A recent study evaluated a combined proinflammatory and immunomodulatory cytokine panel including IFNγ to predict viral load set point 12 months after infection (23). In the combined panel, IFNγ, IL-12p40/70, IL-7, and IL-15 levels predicted 66% of viral set point variation in acute phase patients (23). Further studies are required to evaluate the predictive value of this panel on morbidity and progression of patients with chronic disease. In addition to the polyfunctional effects of IFNγ, and due to the marked complexity of the antiviral response, it is likely that monitoring of HIV-1 patients by a proinflammatory cytokine panel rather than relying on a single cytokine will better predict viral load set point and progression of clinical disease.

## IFNγ in Therapy Against HIV/AIDS

Many studies have been performed to determine the roles that IFNγ plays in anti-HIV therapy. Initially, clinical studies determined that IFNγ can either hinder or augment the pathogenesis of HIV-1. The latter observations raised a major concern about the use of IFNγ in HIV-1 therapy. Concomitantly, a number of *in vitro* studies tested the anti-HIV activity of IFNγ on HIV-1 infection but with conflicting results. Subsequently, a small number of clinical trials investigated whether IFNγ has therapeutic effects against HIV-1 in HIV^+^ subjects. With the growing understanding of the roles that cytokines play in infection and disease progression, cytokines including IFNγ have been measured to assess the efficacy of anti-retroviral therapy (ART). ART has greatly improved the quality of life and the lifespan of the HIV-infected subjects but does not substantially restore the immune system destroyed by HIV-1. Consequently, IFNγ and cytokines which induce or enhance IFNγ activity have been considered for therapy to restore the immune system in particular T cell number and function. The opposing or conflicting effects of IFNγ on HIV-1 pathogenesis and immune function have complicated the role that IFNγ plays on anti-HIV therapy.

### IFN antiviral activity

Interferons were originally discovered, named, and characterized based on their ability to inhibit viral replication (24). These soluble factors are classified as Type I and II IFNs with IFNγ being the sole representative of the Type II IFN family (25, 26). Although both Type I and II IFNs can induce an antiviral host response, they differ by both antigenic induction, receptor specificity, and cell expression. While Type I IFNs are largely induced by viral infection of host cells, IFNγ is induced by more generalized antigenic and mitogenic stimulation (25). Type I IFNs are secreted at low levels by almost all cell types, however are primarily secreted by hematopoietic cells (IFNα, IFNω) and fibroblasts (IFNβ) (26). IFNγ is primarily produced by CD4^+^ and CD8^+^ T cells as well as NK cells with more recent reports of low level expression in NKT cell and professional antigen presenting cells (26). Both Type I and II IFNs induce a wide range of proteins with activity targeting different stages of viral replication. However, IFNγ upregulates MHC-I on the cell surface, which increases antigenic recognition of intracellular pathogens by CTLs. In addition, only IFNγ can upregulate the MHC-II pathway, supporting antigen-specific activation of CD4^+^ T cells (25, 26).

There are a number of IFN-induced proteins and gene products that confer antiviral activity. The first of these is dsRNA-regulated protein kinase (PKR) which is a serine/threonine kinase found predominantly in the cytoplasm and associated with ribosomes. PKR is activated by dsRNA and inhibits the synthesis of viral proteins through phosphorylation of eukaryotic translation initiation factor-2 (eIF-2). In addition to antiviral activity, PKR also plays a role in modulation of cell proliferation and induction of apoptosis (25, 26). The dsRNA-specific adenosine deaminase (ADAR), catalyzes the deamination of adenosine to inosine, resulting “editing” or mistranslation of the viral sequence. Mistranslation of gene products can lead to the production of non-functional viral proteins. The 2′,5′-oligoadenylate synthetase (OAS) in combination with RNase L is activated by dsRNA during viral infection and induces degradation of RNA. The protein Mx GTPases, a superfamily of dyamin-like GTPases, associate with viral protein complexes to impair transport of viral nucleocapsids into the nucleus of the host cell, preventing transcription. Type 1 IFN-regulated gene expression of Mx1 and CD317 may be involved in control of HIV neurovirulence (27). Although Mx GTPases are induced by Type I IFNs but not by Type II IFNγ (25), other classes of GTPases are induced by IFNγ allowing antiviral targeting of GTP by other mechanisms (26). More recent findings on IFN-induced tetherin/BST-2, an antagonist of HIV Vpr, may be important in prevention/control of HIV infection via innate immunity (28).

Several IFN-inducible mechanisms are involved in host response and immune evasion of HIV. The expression of HIV Trans-activator of transcription (Tat) can either negatively or positively affect IFN-induced PKR in regulating HIV-1 infection. Tat can prevent autophosphorylation of PKR and competes with eIF-2 while upregulating NF-κB to promote transcription (25). IFN response/regulatory factors (IRFs) compete with the binding site of HIV’s LTR promoter and suppress viral transcription (29). Both Type I and II IFNs induce PKR, OAS, and ADAR, however there is a significant difference in the sensitivity to Type I and II IFNs to HIV in PBMC, T cells, and macrophages. Although the specific cause has not been elucidated, it is likely that the constant presence of IFNγ will most likely promote negative regulation of IFNγ signaling through the SOCS pathway (particularly SOCS1) and PIAS, a Stat inhibitor. Induction of these proteins target at various stages of viral replication and can induce an “antiviral state” within the host. However, adaptation and host evasion mechanisms allow replication of species adapted lentiviruses [HIV in humans, simian immunodeficiency virus (SIV) in macaques] despite the induction of IFN-induced anti-retroviral states (30).

### The use of IFNγ in HIV/AIDS therapy

The direct effect of IFNγ on HIV-1 infection was first evaluated in *in vitro* studies followed by small scale clinical trials. *In vitro* studies have shown IFNγ treatment to either enhance or have no effect on HIV-1 infection of PBMC (31). These observations with PBMC were strikingly different from what was expected in 1986 since all interferons (α, β, γ) were thought to have direct antiviral activities to all types of viruses (32). Subsequent, *in vitro* studies demonstrated that IFNγ treatment can enhance HIV-1 infection in both primary macrophages and CD4^+^ T cells (33–35), suggesting that these immune cell subsets were responsible for the original observation of HIV-1 infection of PBMC.

Given that IFNγ is also produced early during cytokine storms in the acute stage of HIV-1 infection, IFNγ was thought to affect the subsequent development of CTL activities to control HIV-1 load (36–38). There is a conflicting view regarding the role of cytokines such as IFNγ in modulating cellular immunity which can in turn determine the viral set point (38, 39), as a high viral set point is positively associated with progression to AIDS (40, 41). The well documented role of IFNγ in enhancing CTL activities against viral infection has supported the concept that IFNγ therapy can augment anti-HIV CTL activities in HIV^+^ subjects (42, 43). This is further supported by the finding that early control of HIV-1 load correlates with production of anti-HIV CD4^+^ and CD8^+^ CTLs (36, 37, 44), while similar control of virus load has been described for HIV^+^ long-term survivors (LTSs) (45), elite controllers (ECs) (46, 47), and highly exposed persistently seronegative women (48). Based on these observations, clinical trials in adult patients have evaluated the toxicity, pharmacokinetics, and therapeutic effect of IFNγ on HIV-1 p24 load, AIDS-associated complex (ARC), and AIDS-associated Kaposi’s sarcoma (KS) (49–52). The majority of the clinical trials showed no significant improvement in ARC or KS as well as no significant decrease in HIV-1 p24 load (Table 2) (49–52). IFNγ had no effect at doses that conferred therapeutic efficacy for IFNα or even at higher doses that resulted in mild toxicity (53–57). Notably, in comparison to the potent anti-HIV and -KS activities of IFNα and β, the lack of anti-HIV/AIDS activities of IFNγ greatly reduced the enthusiasm toward IFNγ and therapeutic focus shifted to IFNα as therapy against HIV/AIDS (57). Although clinical trials have also been conducted with pegylated IFNα to modulate its activity, the general consensus is that IFNα therapy is too toxic and significantly less effective than ART in decreasing HIV-1 load but is effective for treatment of KS (58–60).

**Table 2:** **Interferon-γ in therapy against HIV/AIDS**.

Description^a^	IFNγ activity or response	Reference
**IFNγ therapy against HIV/AIDS**	No effect on HIV load, CD4^+^ T cell count, and disease progression	(53–57)
**Adjunctive IFNγ therapy for opportunistic infection**	Decreasing trend to significant decrease in opportunistic infection	(61–67)
**IFNγ levels during ART**
Serum IFNγ levels
Cross-sectional study	Decreasing trend in serum IFNγ	(20)
Longitudinal study	Significant decrease in serum IFNγ	(20)
T cell responses
CD4^+^ T cells	Varying IFNγ responses	(68–70)
CD8^+^ T cells	Generally decreasing IFNγ response	(70, 71)
Polyfunctional CD8^+^ T cells	Gradual increase in IFNγ response with prolonged ART	(71)

*^a^Chronically HIV-1 infected subjects*.

### IFNγ levels at pre- and post-ART

Clinical assessment of IFNγ levels in the serum of HIV^+^ adult subjects at different clinical stages has been used to determine the importance of IFNγ in the pathogenesis of HIV-1. Clinical studies have focused on the changes in cytokine levels upon introduction of highly active anti-retroviral therapy (HAART), or ART. The elevation of multifunctional cytokines such as IFNγ and TNFα, can either enhance or control HIV-1 infection depending on the clinical stage of HIV-1 infection. A cross-sectional study showed significantly elevated serum levels of certain cytokines (TNFα, IL-6, IL-7, IL-10, IL-18) and an increasing trend for serum IFNγ levels in symptomatic subjects when compared to asymptomatic subjects prior to treatment with ART (20). Cross sectional and longitudinal clinical studies, comparing pre-ART to post-ART subjects demonstrated high serum levels of many cytokines (IL-6, IL-10, IL-18) in pre-ART subjects which significantly decreased when ART was initiated. Concomitant with ART, the serum HIV-1 load decreased to low or undetectable levels while the CD4^+^ counts and serum IL-21 often increased (20, 72–77). In the case of IFNγ, cross-sectional study of pre- and post-ART showed a decreasing trend in serum IFNγ levels with the initiation of ART (Table 2) (20). In comparison, a longitudinal study showed a statistically significant decrease in IFNγ after 60 days or longer on ART (20). Although the majority of the subjects had a major decrease in IFNγ, 33% of the subjects maintained high serum IFNγ levels. In this study, all HIV^+^ subjects were treated for secondary clinical diseases before enrollment to ensure cytokine changes during the study were predominantly attributed to HIV-1 infection. Thus, the authors of this work speculated that sustained high IFNγ levels in this group were due to individual differences in immune responses against HIV-1, the genetic characteristics of HIV-1, or both, and not due to other potentially confounding clinical events (20).

Another approach for evaluating the immune status at pre- and post-ART is to measure the level of IFNγ responses to HIV-1 proteins or peptides by the PBMC or T cells from HIV^+^ subjects (78, 79). The hallmark of HIV-1 infection is the loss of CD3^+^CD4^+^ T cell counts correlating with increases in both virus load and disease progression (80). As a result, measuring T cell immunity, specifically CD3^+^CD4^+^ T cell activities, was thought to be useful at assessing the immune status of the HIV^+^ subject when analyzed in combination with CD3^+^CD4^+^ T cell count and virus load (81, 82). HIV-specific CD3^+^CD8^+^ T cell activities develop shortly after the cytokine storm and work to control HIV-1 load during acute infection (37–39). Remarkably, IFNγ responses were consistently detected in both CD3^+^CD4^+^ and CD3^+^CD8^+^ T cells of the HIV^+^ subjects at various clinical stages, but IFNγ responses alone had no direct correlation to delay in progression to AIDS (78, 82–84). The presence of polyfunctional T cells, which expressed IFNγ in combination with other cytokines (IL-2 and TNFα) and/or cytotoxins (perforin or granzyme), was associated with HIV-1 non-progression (81–84). CD3^+^CD4^+^ and CD3^+^CD8^+^ T cells induced with viral epitopes are important effector cells against HIV-1 infection. The IFNγ analysis of T cells from chronically HIV-1 infected patients during ART demonstrated that HIV-specific IFNγ responses varied within the T cell subsets evaluated (68–71, 85). Moreover, during ART, IFNγ responses of HIV-specific CD4^+^ T cells expanded and contracted (68), decreased (69), or increased (70); while those of CD8^+^ T cells generally decreased (70, 71) (Table 2). Interestingly, HIV-specific IFNγ responses of polyfunctional CD8^+^ T cells increased (85).

### IFNγ as an adjunctive cytokine therapy

The highly effective ART was released in developed countries in late 1990s (86) and in developing/underdeveloped countries in mid 2000 (87). ART is a combination of two or more anti-retroviral drugs that inhibits viral reverse transcriptase (RT) (nucleoside and non-nucleoside RT inhibitors), protease, integrase, viral co-receptor attachment (CCR5 inhibitor), and/or virus penetration (fusion inhibitor) (88). ART will decrease the circulating HIV-1 load to low or undetectable levels in plasma within weeks to months (89). It has dramatically reduced the HIV-associated morbidity and mortality but the opportunistic infections and AIDS-associated cancers still persist despite ART (88). Moreover, even after 7–10 years of ART and viral control, a complete reconstitution of immune responses to HIV-1 has not been achieved while only a modest improvement in HIV-specific T cell responses was observed (90–92). Consequently, a rapid means to restore anti-HIV T cell immunity is still required.

Many therapy using cytokines (IL-2, IL-12, G-CSF, GM-CSF) including IFNγ have been evaluated in combination with ART as immune reconstitution therapy (93–95). These therapies are needed due to the new-onset opportunistic infections resulting from failed ART combinations or simply due to the inability of ART to completely eliminate the opportunistic infection (96–98). In one study in South Africa, tuberculosis (TB) incidence rates during 8 years of follow-up showed substantially higher rates in HIV^+^ subjects on long-term ART than in HIV uninfected individuals living in the same community (92). IFNγ has been used as adjunctive immunotherapy with or without ART for the treatment of HIV-associated opportunistic infections such as cryptococcal meningitis (61–63), *Pneumocystis carinii* (64, 65), *Toxoplasma gondii* (65), *Candida albicans* (64, 65), *Mycobacterium avium* (65, 66), and visceral leishmaniasis (65, 67). In a majority of the cases, adjunctive IFNγ therapy with or without other cytokines did not adversely affect the ART therapy for those on ART (i.e., maintained low to undetectable virus load) and did not increase CD4^+^ T cell counts in most HIV^+^ patients except for those ART (61–66). These therapies had either a decreasing trend or a significant decrease in various HIV-associated opportunistic infections which were often resistant to conventional therapy against the organism (Table 2) (61–67).

Ten to 32% of AIDS patients starting ART develop an unusual disease condition called immune restoration disease (IRD) or immune reconstitution inflammatory syndrome (IRIS) (99–103). IRIS is a disease condition where the opportunistic infections or other diseases (e.g., Graves’ disease, neoplasm, or virus-associated diseases) of the AIDS patients worsen shortly after the initiation of ART (99–101). The neoplasm and/or virus-associated diseases observed in IRIS included KS with human herpesvirus-8, non-Hodgkin’s lymphoma with Epstein–Barr virus, and progressive multifocal leukoencephalopathy with JC virus (99, 101). IFNγ together with TNFα, C-reactive protein, and IL-7 are the inflammatory cytokines all contribute to the development of IRIS (101–103). As a result, anti-inflammatory therapy in addition to the anti-microbial therapy, is commonly used to treat IRIS associated with opportunistic infections (99).

## IFNγ in the Development of an Effective HIV-1 Vaccine

The development of an effective HIV-1 vaccine for humans requires the identification of protective HIV-1 vaccine epitopes conserved among most HIV-1 subtypes, the construction of protective epitopes into a vaccine immunogen, and determining the best vaccine delivery system for induction of both mucosal and systemic immunity against HIV-1. As the cytokines expressed by many T cell subsets, IFNγ and IL-2 have been used as the biomarkers for CD4^+^ and CD8^+^ T cell activities induced by candidate HIV-1 vaccine antigens. Both of these cytokines are important in enhancing HIV-specific CTL activities and antibody synthesis essential for generating vaccine immunity. Ideally, cytokines produced by CD3^+^CD4^+^ Th cells should augment effector functions of both T and B cells upon vaccination. Initial HIV-1 vaccine studies searched for B-cell epitopes on HIV-1 envelopes (transmembrane and surface envelopes) that induced broadly reactive virus neutralizing antibodies, while subsequent vaccine studies focused on developing an HIV-1 vaccine that induced potent anti-HIV CTL activities. IFNγ has had a major impact on determining the presence of CD8^+^ CTL epitopes on HIV-1 proteins, with the highest levels detected from HIV-1 Gag, Pol, and Nef proteins. In the most successful HIV-1 vaccine trial, both polyfunctional CD4^+^ T cells and CD4^+^ CTLs that expressed IFNγ and other cytokines or cytotoxins were detected in the vaccinees. Thus far, IFNγ has played a major role as a biomarker of T cell activation in the development of an HIV-1 vaccine.

### IFNγ in evaluating HIV vaccine epitopes

The IFNγ responses to HIV-1 peptides by the PBMC or T cells from HIV^+^ subjects have been used to identify the regions on the virus that induced CD3^+^CD8^+^ CTL and CD3^+^CD4^+^ T cell activity (78). Three of the most commonly used assays for such analysis are tetramer staining, IFNγ ELISpot analysis, and FACS-based intracellular staining (ICS) for IFNγ in combination with T cell phenotypic markers (16, 78, 104, 105). Since the reagents for IFNγ became available before other cytokines and cytotoxins, the latter two analyses frequently utilize IFNγ. Furthermore, IFNγ responses of T cells are detected throughout the duration of HIV-1 infection (78, 106). IFNγ ELISpot is a more rapid and cost efficient assay than ICS or tetramer analyses. The IFNγ ELISpot analysis using purified CD3^+^CD8^+^ and CD3^+^CD4^+^ T cells was initially used to map the CD8^+^ CTL and CD4^+^ Th epitopes on HIV-1 proteins. Some of the HIV-1 epitopes defined by IFNγ ELISpot analysis has been confirmed by IFNγ-specific ICS (104, 105). The CTL and TH epitopes on all HIV-1 proteins have been listed in Los Alamos National Laboratory (LANL) database (http://www.hiv.lanl.gov/content/immunology/maps/maps.html). Such database is useful in identifying the HIV-1 epitopes needed for developing prophylactic vaccines as well as immune-based therapy against HIV-1.

Perhaps the most problematic issue with the use of IFNγ-based analysis is that the IFNγ levels from CD4^+^ and CD8^+^ T cells alone do not correlate with HIV-1 load or disease progression (Table 3) (78, 79, 83, 105, 107). For this reason, the HIV-specific IFNγ levels only indicate the ability of these T cell subsets to produce IFNγ responses to HIV-1 peptides. Polyfunctional T cells, which are involved in controlling HIV-1 load, express IFNγ in addition to other cytokines and cytotoxins (48, 49, 81–84) particularly in a combination of IFNγ with perforin, IL-2, TNFα, or granzyme B. These T cells are thought to be important in the control of HIV-1 infection in HIV^+^ LTSs and ECs (46, 81, 82). In addition, proliferative T cells expressing IFNγ have also been observed at higher levels in LTS and EC, but at lower levels in HIV^+^ progressors (46, 82). These studies suggest that vaccines that only induce IFNγ in HIV-specific CD4^+^ T cells and/or CD8^+^ T cells are unlikely to protect humans against HIV-1. Instead, those that induce polyfunctional T cell activities against HIV-1 are more likely to be useful as vaccine immunogens.

**Table 3:** **Interferon-γ in development of an HIV-1 vaccine**.

Description	IFNγ activity or immune response	Reference
**Identifying vaccine epitopes^a^**	Epitopes inducing only IFNγ do not correlate with HIV load or disease progression	(78, 79, 105, 107)
**Cytokine adjuvant^b^**
Genetic IFNγ adjuvant for DNA vaccine	No effect or some enhanced DNA vaccine efficacy in animal models	(108 –111)
IFNγ adjuvant for protein-based vaccine	No effect in an animal model	Pu and Yamamoto, unpublished observation
**Phase IIb–III vaccine trials**
Phase III VaxGen 003 and 004 trials	IFNγ responses by CD8^+^ T cells	(112)
Phase IIb STEP trial	IFNγ responses by T cells	(113)
Phase III RV144 trial	IgG antibodies to Env-V1V2 inversely correlate with HIV infection rate	(114)
	IFNγ and/or IL-2 responses to Env by CD4^+^ T cells	(115)
	IFNγ, IL-2, and/or TNFα responses to Env by polyfunctional CD4^+^ T cells	(116)

*^a^Evaluation in long-term survivors, elite controllers, and progressors*.

*^b^SIV/macaque model in genetic adjuvant and FIV/cat model in genetic and protein adjuvants*.

### IFNγ in preclinical vaccine trials

Preclinical trials in the SIV/macaques model demonstrate the induction of SIV-specific polyfunctional CD4^+^ and CD8^+^ T cell activity resulting in marked decreases in viral load using DNA or viral vector vaccine strategy as either post-infection therapy or prophylaxis against SIV (117–120). Epidermal co-delivery of SIV Gag, RT, Nef, and envelope (Env) DNA vaccine formulated with a mucosal genetic adjuvant induced a substantial decrease in peripheral and mucosal viral burden in chronically infected macaques (117). Durable and sustained suppression of viral load and non-progression was positively associated with significant increases in SIV-specific IFNγ responses in T cells from peripheral blood (117, 118) and gut mucosa (117). In addition, vaccination induced SIV-specific CD8^+^ T cells with dual TNF-α and cytolytic effector functions in peripheral blood (117). In vectored vaccine studies, rhesus cytomegalovirus vector expressing HIV-1 Gag, Pol, Nef, Env, RT, and integrase sequences demonstrated a high frequency of CD69^+^IFNγ^+^ and/or CD69^+^TNFα^+^ CD4^+^ and CD8^+^ effector memory T cell response that correlated to control of highly pathogenic SIV_mac239_ infection after mucosal challenge (119). The suppression of peak viral load and protection from challenge correlated with the magnitude of the peak SIV-specific CD8^+^ T cell responses in the acute phase post-vaccination (described as the vaccine phase). Thirteen of the 24 vaccinated macaques had undetectable plasma viral loads that persisted up to 1 year (119). These responses have been demonstrated in distinct MHC-I and -II restricted CD8^+^ T cells, suggesting distinct patterns of epitope recognition in these T cell subsets (120). These studies outline the importance of the polyfunctional T cell responses in suppression of viral load in chronic infection and prevention of virus rebound after cessation of ART, and in the prophylaxis against AIDS viruses.

### IFNγ as a cytokine adjuvant for HIV-1 vaccine

The use of IFNγ as cytokine adjuvant for HIV-1 vaccines has been studied more extensively in animal models of AIDS than in humans. The rationale for using IFNγ is based on its ability to promote CTL and NK cell activities as well as antibody production including induction of isotype switching (6, 42, 43, 121–124). Many cytokines, including those of IFNγ and IFNγ-inducing cytokine (IL-12), have been used as a genetic-based cytokine adjuvant to enhance lentiviral DNA vaccines (108, 125). SIV/macaque and feline immunodeficiency virus (FIV)/cat AIDS models have been used extensively to determine the effect of cytokine as an adjuvant (Table 3) (108–111, 125). In one study, a vaccine consisting of SIV and IFNγ DNA constructs was more effective against challenge with heterologous SIV than SIV DNA vaccine alone (109). However, a later SIV/macaque study was unable to confirm the results from the initial study (108). Furthermore, in FIV/cat model, laboratory cats vaccinated with FIV proviral deletion mutant (Δvif or ΔRT) and IFNγ DNA construct conferred either no protection (FIV_Δvif_/IFNγ) or marginal protection (FIV_ΔRT_/IFNγ) against heterologous FIV challenge when compared to cats vaccinated with FIV DNA construct alone (110, 111).

Cytokine adjuvant is more commonly used in DNA vaccines to enhance the low immune responses generated by the low viral antigen expression of the viral DNA (126, 127). Nevertheless, cytokine-adjuvant studies have been performed with protein-based FIV vaccines containing conventional adjuvant. These studies show no (IFNγ and IL-18) to moderate (IL-12 and -15) enhancement of protective activity of the viral immunogen or inactive whole-virus FIV when compared to those without cytokine (Table 3) (128, 129). In one study, laboratory cats immunized with inactivated FIV vaccine supplemented with IFNγ in conventional adjuvant did not augment the protection observed with the vaccine without IFNγ (Pu and Yamamoto, unpublished observation). The inability of IFNγ to enhance the vaccine efficacy may be attributed to the fact that AIDS lentiviral proteins themselves can induce IFNγ production in T cells as observed in animals vaccinated with viral protein or inactivated virus in conventional adjuvant (128, 130–133). Similarly, many of the HIV-1 proteins and peptides (core p24, enzyme RT, accessory Nef, and envelope gp120) can directly stimulate T cells from HIV^+^ subjects or vaccinated HIV-negative subjects to produce IFNγ (78, 79, 133–135). For these reasons, the use of IFNγ as a cytokine adjuvant is unlikely to enhance the activities of an HIV-1 protein vaccine.

### IFNγ in phase IIb–III HIV-1 vaccine trials

The last four major HIV-1 vaccine trials in humans consisted of two phase III vaccine trials using recombinant HIV-1 envelope gp120 protein of subtype B (VaxGen 004 trial) and subtypes B and E combined (VacGen 003) (135, 136); phase IIb STEP trial using adenovirus-5 vector (Ad5) expressing subtype-B HIV-1 *gag/pol/nef* (137); and phase III RV144 prime-boost trial consisting of canarypox vectored HIV-1 *gag/pr/gp41–120* priming and boosting with subtypes B and E recombinant gp120 proteins (114, 115). More importantly, among the four trials only the prime-boost RV144 trial had some efficacy. The RV144 trial had an efficacy of 31.2% in a general population but the efficacy of only 3.7% in the high risk group. In contrast, VaxGen gp120 trials had neither efficacy nor adverse effects (135, 136), while the STEP Ad5-vectored *gag/pol/nef* trial showed more HIV-1 infection in vaccinated subjects than placebo immunized subjects (137). All of the above vaccines induced HIV-specific IFNγ responses from either CD4^+^ or CD8^+^ T cells and the duration of IFNγ expression varied between the trials (Table 3) (112, 113, 116, 138).

In the initial report on RV144 trial, the CD4^+^ and CD8^+^ T cells of the vaccinees were evaluated for IFNγ ELISpot and IFNγ/IL-2-specific ICS responses to HIV-1 Gag and Env (115). Only IFNγ/IL-2-specific ICS to Env in the CD4^+^ T cells were significantly higher (*p* < 0.001) in the vaccinated group than in the placebo group (Table 3) (115). A more extensive immune-correlated analysis of RV144 trial demonstrated that the binding of IgG antibodies to variable regions 1 and 2 (V1, V2) of HIV-1 Env inversely correlated with the rate of HIV-1 infection (*p* = 0.02) (114). In contrast, the binding of plasma IgA antibodies to Env correlated positively with the rate of HIV-1 infection. Moreover, HIV-1 neutralizing antibodies, T cell responses, and specifically T cell produced IFNγ responses that were detected in the vaccinees did not significantly affect the HIV-1 infection rate (114). A more extensive analysis of T cell activity indicated that the prime-boost vaccination induced polyfunctional (IFNγ^+^, IL-2^+^, and/or TNFα^+^) and potentially cytolytic (cytolytic marker CD107a^+^) CD4^+^ T cell responses to HIV-1 Env peptides, including the V2 peptides, 6 months after the last immunization (116). Thus, the most promising RV144 trial demonstrated the importance of IFNγ in detecting polyfunctional T cells.

## Conclusion

The difficulty in correlating serum IFNγ levels with HIV/AIDS clinical status has been attributed to the role that IFNγ plays as an inflammatory cytokine as well as a cytokine that enhances antiviral immunity. During the acute stage of HIV-1 infection, the host immune system mounts an inflammatory response resulting in a cytokine storm. In the cytokine storm, a number of inflammatory cytokines including IFNγ are produced which decrease as the adaptive immune responses against HIV-1 develop. If not appropriately controlled, such inflammatory activities can enhance HIV-1 infection and may cause a higher viral set point before T cell immunity can control the HIV-1 load. Remarkably, low levels of IFNγ are detected throughout the course of HIV-1 infection correlating with persistently increasing HIV-1 load. Furthermore, many of the HIV-1 proteins can directly stimulate T cells from HIV^+^ subjects to produce IFNγ, leading to chronic immune activation and ultimately the exhaustion of the immune system and resulting in the loss of IFNγ production.

Interferon-γ therapy had no effect on HIV-1 load or AIDS progression whereas ART had a dramatic effect on both. However, long-term ART did not completely restore the immune responses in HIV-1 or completely eradicate the opportunistic infections. As a result, IFNγ alone or in combination with other cytokines has successfully been used together with ART against HIV-associated opportunistic infections. Lastly, polyfunctional CD4^+^ T cells that expressed IFNγ were observed in the vaccines of the most effective HIV-1 vaccine (RV144) trial to date. Hence, IFNγ may still play an important role as a product of HIV-specific polyfunctional CD4^+^ T cells which may serve to enhance the anti-HIV antibody production as well as CTLs against HIV-1.

## Conflict of Interest Statement

The authors declare that the research was conducted in the absence of any commercial or financial relationships that could be construed as a potential conflict of interest.
